# Supplementary material for: Association between Dietary Patterns and Low HDL-C among Community-Dwelling Elders in North China
Source: Nutrients. 2021 Sep 22;13(10):3308. doi: 10.3390/nu13103308 (PMC8537052; doi:10.3390/nu13103308)
Supplement: Supplementary file 1 [file nutrients-13-03308-s001.zip › nutrients-1354165-supplementary.pdf]

Supplemental Table S1. Pearson correlation among significant variables in the elder participants in North China (N=3,387).

| | | **Age** | **BMI** | **PA** | **WC** | **TC** | **TG** | **HDL-C** | **LDL-C** | **SBP** | **DBP** |
|---|---|---|---|---|---|---|---|---|---|---|---|
| **Age** | **Coef.** | 1.000 | -0.084 | -0.192 | 0.020 | -0.059 | -0.052 | -0.037 | -0.051 | 0.104 | -0.111 |
| | *P value* | | <.0001 | <.0001 | 0.253 | 0.001 | 0.003 | 0.032 | 0.003 | <.0001 | <.0001 |
| BMI | **Coef.** | -0.084 | 1.000 | -0.073 | 0.679 | 0.127 | 0.246 | -0.241 | 0.204 | 0.181 | 0.192 |
| | *P value* | <.0001 | | <.0001 | <.0001 | <.0001 | <.0001 | <.0001 | <.0001 | <.0001 | <.0001 |
| PA | **Coef.** | -0.192 | -0.073 | 1.000 | -0.094 | -0.037 | -0.064 | 0.059 | -0.047 | -0.055 | -0.039 |
| | *P value* | <.0001 | <.0001 | | <.0001 | 0.031 | 0.000 | 0.001 | 0.006 | 0.001 | 0.025 |
| WC | **Coef.** | 0.020 | 0.679 | -0.094 | 1.000 | 0.099 | 0.222 | -0.259 | 0.169 | 0.180 | 0.173 |
| | *P value* | 0.253 | <.0001 | <.0001 | | <.0001 | <.0001 | <.0001 | <.0001 | <.0001 | <.0001 |
| **TC** | **Coef.** | -0.059 | 0.127 | -0.037 | 0.099 | 1.000 | 0.295 | 0.313 | 0.931 | 0.101 | 0.063 |
| | *P value* | 0.001 | <.0001 | 0.031 | <.0001 | | <.0001 | <.0001 | <.0001 | <.0001 | 0.000 |
| **TG** | **Coef.** | -0.052 | 0.246 | -0.064 | 0.222 | 0.295 | 1.000 | -0.380 | 0.272 | 0.094 | 0.054 |
| | *P value* | 0.003 | <.0001 | 0.000 | <.0001 | <.0001 | | <.0001 | <.0001 | <.0001 | 0.002 |
| **HDL-C** | **Coef.** | -0.037 | -0.241 | 0.059 | -0.259 | 0.313 | -0.380 | 1.000 | 0.072 | -0.005 | -0.021 |
| | *P value* | 0.032 | <.0001 | 0.001 | <.0001 | <.0001 | <.0001 | | <.0001 | 0.751 | 0.215 |
| **LDL-C** | **Coef.** | -0.051 | 0.204 | -0.047 | 0.169 | 0.931 | 0.272 | 0.072 | 1.000 | 0.089 | 0.071 |
| | *P value* | 0.003 | <.0001 | 0.006 | <.0001 | <.0001 | <.0001 | <.0001 | | <.0001 | <.0001 |
| **SBP** | **Coef.** | 0.104 | 0.181 | -0.055 | 0.180 | 0.101 | 0.094 | -0.005 | 0.089 | 1.000 | 0.551 |
| | *P value* | <.0001 | <.0001 | 0.001 | <.0001 | <.0001 | <.0001 | 0.751 | <.0001 | | <.0001 |
| **DBP** | **Coef.** | -0.111 | 0.192 | -0.039 | 0.173 | 0.063 | 0.054 | -0.021 | 0.071 | 0.551 | 1.000 |
| | *P value* | <.0001 | <.0001 | 0.025 | <.0001 | 0.000 | 0.002 | 0.215 | <.0001 | <.0001 | |

TC: total cholesterol; TG: triglyceride; HDL-C: high-density lipoprotein cholesterol; LDL-C: low-density lipoprotein cholesterol; BMI: body mass index; WC: waist circumference; PA: physical activity of median and high intensity (minutes per week)
